# Supplementary material for: Continuous Intracranial Pressure Monitoring in Children with ‘Benign’ External Hydrocephalus
Source: J Clin Med. 2025 Apr 28;14(9):3042. doi: 10.3390/jcm14093042 (PMC12072479; doi:10.3390/jcm14093042)
Supplement: Supplementary file 1 [file jcm-14-03042-s001.zip › jcm-3538776-supplementary.pdf]

# Supplementary Material

## Complete information on the methodology for ICP monitoring

Continuous ICP was performed for at least 48 hours in each patient using an extradural sensor (Neurodur-P, Raumedic, Rehau AG+Co, Rehau, Germany). The Neurodur-P sensor is specifically designed for continuous extradural ICP monitoring and includes a microchip precision sensor at the catheter tip [32]. The ICP sensor was implanted through a 12 mm burr hole under general anesthesia in the pre-coronal region of, usually, the left hemisphere at 3 - 4 cm of the midline. Special attention is always paid to the circumferential dissection of the epidural space: approximately 20 mm in a forward direction from the burr hole in the anterior 180º arc and approximately 10-15 mm in the posterior 180º arc [32]. The sensitive side of the sensor is applied against the dura mater, and the ICP sensor is connected to a Raumedic monitor (MPR2 logO DATALOGGER, Rehau AG+Co, Rehau, Germany). Apart from the digital or paper recording, the end-hour ICP is always recorded by the nurse in charge. In the first four children in the study, hard copies of the ICP values were obtained using an analog pen recorder (Yokogawa 3021 Pen Recorder, ADLER S.A., Madrid) at a paper speed of 20 or 60 cm/hour. All other patients were monitored utilizing a custom-designed digital recording technology platform based on the ADInstruments PowerLab 4SP hardware and LabChart v8.1 software (ADInstruments, Ltd., Grove House, Hastings, UK) with the ICP signal sampled at 200 Hz, well above the minimum rate at which digital sampling can accurately record an analog signal (Nyquist Frequency) [33]. When converting ICP from the analog form into a digital format, frequency errors can arise due to 'aliasing', a phenomenon that occurs when a dynamic event is sampled at a rate insufficient to represent its behavior accurately. At a sampling rate of 200 Hz, the characteristic cardiac waveform, its amplitude, and the three sub-components of the wave (P1, P2, and P3) are preserved and can be analyzed if needed [33].

ICP monitoring was performed for at least 48 hours, including at least two overnight recordings. All recorded data were stored on a laptop computer running Windows 10 (Microsoft Windows, Redmond, USA). To reduce signal noise and enhance the detection of slow waves in raw ICP recordings, a second digital channel was created. This channel utilized a smoothing triangular Bartlett digital filter, implemented in the LabChart software with a window length of 255 samples, to produce a time-domain

smoothed signal. This approach allowed for clearer visualization and analysis of slow-wave activity in the ICP data, improving the accuracy and reliability of the recordings while minimizing the impact of high-frequency noise. This filter, similar to the Savitzky-Golay filter used by Riedel et al. [34], reduces the effects of aliasing, and is a good replacement for a traditional moving average [34,35] (**Fig. 2, main article**). For quantitative analysis of the ICP recordings, one of the two senior authors (MAP or JS) reviewed all ICP recordings using LabChart v8.1 and extracted the relevant metrics. Mean ICP: The mean ICP corresponding to the total recording period was calculated manually using the LabChart 'data pad'. Data obtained from 08:00 to 22:00 were used to calculate the 'diurnal' mean ICP. Data obtained from 22:01 to 07:59 were used to calculate 'nocturnal' mean ICP. Definition of slow waves: The presence of slow ICP waves (A- and B-waves) was also quantified according to Lundberg's original definition [36]. For B-waves, we increased the upper frequency to 3 waves/min [37]. Therefore, B-waves were defined as waves with a frequency of 0.5 - 3 waves/min, lasting for at least 10 minutes [36-38]. B-waves were subdivided according to amplitude into high-amplitude B-waves (≥ 10 mmHg) and low-amplitude B-waves (< 10 mmHg). The total sum of high- and low-amplitude B-waves was expressed as the percentage of total monitoring time. Lundberg A-waves were defined as ICP elevations at least 20 mm Hg above the resting line, with abrupt onset and end, lasting between 5 and 20 minutes [36]. The nocturnal rate of pressure waves was calculated by dividing the total duration of pressure waves by the 10-hour night sleep period (from 22:01 to 07:59).

ICP pulse amplitude: We used a modification of the method described by Eide et al. [39,40] to calculate the pulsatility of the cardiac wave ($ICP_{AMP}$) in the time domain. $ICP_{AMP}$ is defined as the difference between diastolic minimum pressure and systolic maximum pressure [39]. The amplitude was calculated using LabChart and a 10-minute time window in two artifact-free selected periods: 1) when the ICP was stable and at its lowest mean ICP without any A- or B-waves (usually during the day) and 2) when the patient presented a train of B- or A-waves, usually during overnight recordings that have a repetitive pattern during REM (Rapid eye movement) sleep [34]. In patients with multiple B- or A-wave trains, we selected the period of time in which the waves had the highest amplitude (**Fig. 3, main article**). This simplified method is equivalent to more complex methods of averaging the $ICP_{AMP}$ during the total monitoring time by using the LabChart predefined settings and allows the operator to select the best artifact-free recording segments (unpublished results). The mean ICP of a 10-minute time window was reported separately as $ICP_{AMP1}$ ($ICP_{AMP}$ in the regular flat recording) and $ICP_{AMP2}$

($ICP_{AMP}$ on top of the B- or A-waves). Following the criteria established by Eide et al., an ICP amplitude ≥ 5 mmHg was considered abnormal and indicative of low intracranial compliance [39,41,42].

## Surgical management protocol for shunt implantation

The surgical management protocol included several peri- and postoperative maneuvers to minimize secondary complications. A prophylactic antibiotic regimen was administered to all patients, consisting of two doses of 20 mg/kg sulfamethoxazole and 4 mg/kg trimethoprim. The first dose was given before anesthesia induction, and the second dose was administered 12 hours later. Additionally, stringent hygiene protocols were followed, including washing the head and body twice: once in the ward before transferring the patients to the operating room and again after the induction of anesthesia. The surgical field was then painted with Betadine solution and covered with Betadine-soaked gauze strips for at least three minutes. These measures were implemented to minimize the risk of infection and ensure optimal surgical conditions. A frontal precoronal position was selected to place the ventricular catheter. The ventricular catheter insertion site in the dura mater was opened using monopolar low-intensity coagulation, and the size of the opening was limited to the diameter of the ventricular catheter. In cases where the ventricles were very small, we utilized ultrasound-assisted insertion to guide the placement of the ventricular catheter (BKActiv 2300-66, GE Healthcare SVH, with the 9063 N11C5s Burr Hole Neuro Transducer). This technique enhanced precision and safety, minimizing the risk of misplacement and ensuring optimal catheter positioning. An intraventricular bolus of vancomycin (20 mg) was administered to all patients to clean the catheter's lumen and prevent infection. When the surgical procedure was finished, moderate abdominal compression was applied using a girdle maintained during the day for 3-4 weeks.

Radiological studies, including shunt X-ray series and CT scans, were performed on all patients before discharge to assess shunt placement and function. Additionally, a follow-up CT scan or MRI was conducted approximately six months post-surgery to monitor ventricular size. Patient outcomes were evaluated by the neurosurgeon in charge at three key time points following shunt placement: at one-two weeks, two months, and six months postoperatively. After this initial period, long-term follow-up

was conducted through annual evaluations to ensure continuous monitoring of neurological status, cognitive and motor development, and shunt function.
